# Recent progress in engineering yeast producers of cellulosic ethanol

**DOI:** 10.1093/femsyr/foaf035

**Published:** 2025-07-04

**Authors:** Roksolana Vasylyshyn, Justyna Ruchala, Kostyantyn Dmytruk, Andriy Sibirny

**Affiliations:** Faculty of Biotechnology, Medical College, University of Rzeszów, Cwiklinskiej 2D, Rzeszów 35-601, Poland; Department of Molecular Genetics and Biotechnology, Institute of Cell Biology, NAS of Ukraine, Drahomanov Street, 14/16, Lviv 79005, Ukraine; Faculty of Biotechnology, Medical College, University of Rzeszów, Cwiklinskiej 2D, Rzeszów 35-601, Poland; Department of Molecular Genetics and Biotechnology, Institute of Cell Biology, NAS of Ukraine, Drahomanov Street, 14/16, Lviv 79005, Ukraine; Faculty of Biotechnology, Medical College, University of Rzeszów, Cwiklinskiej 2D, Rzeszów 35-601, Poland; Department of Molecular Genetics and Biotechnology, Institute of Cell Biology, NAS of Ukraine, Drahomanov Street, 14/16, Lviv 79005, Ukraine

**Keywords:** second-generation ethanol, xylose, fermentation, *Saccharomyces cerevisiae*, *Scheffersomyces stipitis*, *Kluyveromyces marxianus*, *Ogataea polymorpha*

## Abstract

The production of second-generation (2 G) bioethanol, a key sector in industrial biotechnology, addresses the demand for sustainable energy by utilizing lignocellulosic biomass. Efficient fermentation of all sugars from lignocellulose hydrolysis is essential to enhance ethanol titers, improve biomass-to-biofuel yields, and lower costs. This review compares the potential of recombinant yeast strains for 2 G bioethanol production, focusing on their ability to metabolize diverse sugars, particularly xylose. *Saccharomyces cerevisiae*, engineered for enhanced pentose and hexose utilization, is compared with the nonconventional yeasts *Scheffersomyces stipitis, Kluyveromyces marxianus*, and *Ogataea polymorpha*. Key factors include sugar assimilation pathways, cofermentation with glucose, oxygen requirements, tolerance to hydrolysate inhibitors, and process temperature. *Saccharomyces cerevisiae* shows high ethanol tolerance but requires genetic modification for xylose use. *Scheffersomyces stipitis* ferments xylose naturally but lacks robustness. *Kluyveromyces marxianus* offers thermotolerance and a broad substrate range with lower ethanol yields, while *O. polymorpha* enables high-temperature fermentation but yields modest ethanol from xylose. The comparative analysis clarifies each yeast’s advantages and limitations, supporting the development of more efficient 2 G bioethanol production strategies. Strain selection must balance ethanol yield, stress tolerance, and temperature adaptability to meet industrial requirements for cost-effective lignocellulosic bioethanol production.

## Teaser

This review explores advances in engineering yeast for cellulosic ethanol production, comparing *Saccharomyces* and nonconventional species in sugar metabolism, fermentation efficiency, and tolerance to lignocellulosic hydrolysate inhibitors.

## Introduction

Global energy and environmental concerns have intensified efforts to produce biofuels from renewable resources as sustainable alternatives to petroleum-based fuels (Broda et al. 2022). Political instability in oil-producing regions and climate-related risks further emphasize the need for domestic, low-emission energy sources (Cavelius et al. 2023). Ethanol remains the leading renewable liquid fuel in the transport sector. Although production declined in 2020 due to the COVID-19 pandemic, it has since rebounded. The USA and Brazil together produce 80% of global ethanol, with the USA output exceeding 56.78 billion liters in both 2021 and 2022 (https://afdc.energy.gov/data/1033, accessed 18 June 2025). Ethanol’s high octane number and heat of vaporization make it suitable for blending with gasoline or use in flex-fuel and high-compression engines (Watanabe et al. 2022). Several techno–economic analyses have shown that achieving ethanol titers of ∼40–50 g/l, yields >90 % of theoretical, and productivities ≥1 g/l/h is typically necessary to approach economic feasibility for lignocellulosic ethanol production (Tao et al. 2013, Konda et al. 2014). These values remain relevant benchmarks, as confirmed by more recent studies (Zheng et al. 2024). Using this model, Tao et al. (2013) showed that the minimum ethanol selling price (MESP) is highly sensitive to feedstock composition, with values ranging from $1.99 to $2.41 per gallon (mean $2.20 ± 0.21) depending on the location and biomass quality. While engineered strains can meet performance thresholds under controlled conditions, further improvements in process integration and feedstock utilization are essential to reduce MESP below $2.00/gallon.

The current review summarizes recent progress in the development of robust and efficient yeast strains for second-generation (2 G) ethanol production from nonconventional feedstocks. The construction of the advanced 2 G ethanol producers is discussed for *Saccharomyces cerevisiae*, which is widely used in industrial bioethanol production but requires genetic modifications to ferment xylose efficiently; *Scheffersomyces (Pichia) stipitis*, one of the best natural xylose fermenters; *Kluyveromyces marxianus*, known for its broad substrate range, high thermotolerance, and fast growth rate, but limited by lower ethanol yields and sensitivity to inhibitors; and *Ogataea (Hansenula) polymorpha*, the most thermotolerant yeast naturally capable of fermenting xylose. Owing to its high ethanol tolerance, *S. cerevisiae* is extensively applied in first-generation bioethanol production, despite lacking the native ability to metabolize xylose. In contrast, *S. stipitis* utilizes both glucose and xylose, though it is less tolerant to ethanol and requires oxygen for growth. *Kluyveromyces marxianus* demonstrates rapid growth and the ability to ferment a wide range of sugars, including lactose and inulin, at elevated temperatures, yet it often produces lower ethanol titers, exhibits sensitivity to lignocellulosic hydrolysate inhibitors and accumulates significant amounts of xylose by-products. *Ogataea polymorpha* can grow and ferment glucose and xylose at temperatures up to 50°C, making it promising for use in the simultaneous saccharification and fermentation (SSF) process. While this yeast also shows relatively high ethanol tolerance, wild-type strains of *O. polymorpha* produce very low amounts of ethanol from xylose. The drawbacks of all these yeasts can, in many cases, be successfully addressed by combining metabolic and evolutionary engineering approaches, along with classical mutation and selection techniques.

## Yeast-based fermentation of lignocellulosic sugars

Lignocellulosic biomass is composed primarily of glucose and xylose, which are the main fermentable sugars produced during hydrolysis. To efficiently produce 2 G ethanol, it is crucial to develop yeast strains capable of fermenting these sugars as well as other by-products such as cellobiose, l-arabinose, mannose, galactose. While the potential for 2 G ethanol production from lignocellulosic substrates is well-established (Saini et al. 2015, Kucharska et al. 2018), the cost of large-scale production remains a barrier. Even small improvements in fermentation efficiency could lead to significant economic benefits. Therefore, this section highlights *S. cerevisiae* for improved pentose and hexose utilization and explores the potential of non-*Saccharomyces* yeasts, such as *S. stipitis, K. marxianus*, and *O. polymorpha*, in enhancing 2 G ethanol yields.

## *Saccharomyces cerevisiae* engineered for efficient pentose and hexose utilization

Wild-type *S. cerevisiae* cannot ferment the pentoses (xylose and l-arabinose), which are major constituents of lignocellulosic hydrolysates, due to the absence of key enzymes initiating pentose catabolism. As xylose is more prevalent than arabinose, most metabolic engineering efforts have focused on enabling xylose fermentation through the heterologous expression of genes involved in xylose catabolism, particularly those encoding cofactor-independent xylose isomerases (XI) that convert xylose to xylulose. Several attempts have been made to successfully express XI from various sources, including *Thermus thermophilus* (Walfridsson et al. 1996), *Clostridium phytofermentans* (Brat et al. 2009), *Bacteroides stercoris* (Ha et al. 2011), the anaerobic fungus *Piromyces sp*. E2 (Karhumaa et al. 2007), and *Orpinomyces sp*. (Peng et al. 2015). Among these, the strain expressing XI from *Bacteroides vulgatus*, after undergoing adaptive evolution, demonstrated the highest xylose conversion and ethanol yield (0.419 g ethanol/g xylose) compared to all other strains (Peng et al. 2015). Additionally, expressing the codon-optimized XI from *C. phytofermentans* in *S. cerevisiae* led to a 46% increase in the specific growth rate on xylose compared to a strain with the nonoptimized gene version (Brat et al. 2009).

**Figure 1.**
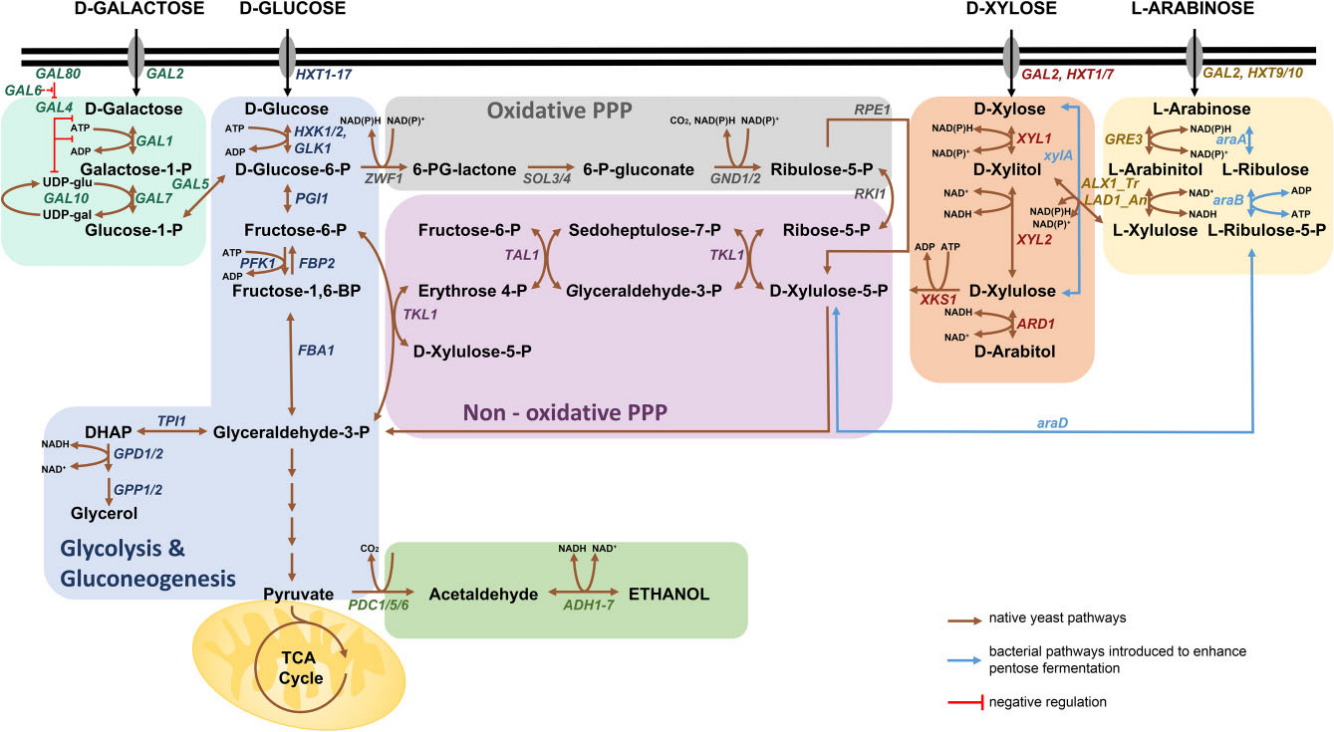
Overview of galactose, glucose, xylose, and l-arabinose metabolic pathways in yeasts. Galactose assimilation pathway: *GAL2*, galactose permease; *GAL1*, galactokinase; *GAL7*, galactose-1-phosphate uridylyltransferase; *GAL10*, UDP-glucose 4-epimerase; *GAL5*, phosphoglucomutase; *GAL80*, repressor of *GAL4; GAL4*, transcriptional activator; and *GAL6*, involved in regulation of *GAL* genes. Glycolysis and gluconeogenesis: *HXK1/HXK2*, hexokinase; *PGI1*, phosphoglucose isomerase; *PFK1/PFK2*, phosphofructokinase; *FBP1*, fructose-1,6-bisphosphatase; *FBA1*, fructose-bisphosphate aldolase; *TPI1*, triosephosphate isomerase; *GPD1/2*, glycerol-3-phosphodehydrogenase; *GPP1/2*, glycerol-3 phosphatase; *PDC1/5/6*, pyruvate decarboxylase; and *ALD1-7*, acetaldehyde dehydrogenase. Oxidative pentose phosphate pathway: *ZWF1*, glucose-6-phosphate dehydrogenase; *SOL3/4*, 6-phosphogluconolactonase; *GND1/GND2*, 6-phosphogluconate dehydrogenase; *RKI1*, ribose-5-phosphate isomerase; and *RPE1*, ribulose-5-phosphate 3-epimerase. Nonoxidative pentose phosphate pathway: *TKL1*, transketolase; *TAL1*, transaldolase. Xylose assimilation pathway: *XYL1*, xylose reductase; *XYL2*, xylitol dehydrogenase; *xylA*, xylose isomerase; and *XKS1*, xylulokinase. l-arabinose assimilation pathway: *GRE3*, aldose reductase from *S. cerevisiae; LAD_An*, l-arabinitol dehydrogenase from *Aspergillus nidulans; ALX_Tr*, l-xylulose reductase from *Trichoderma reesei* (fungal pathway); *araA*, l-arabinose isomerase; *araB*, ribulokinase; and *araD*, l-ribulose-5-phosphate 4-epimerase (bacterial pathway).

Research has focused on creation of oxidoreductive pathway of xylose utilization due to expressing genes for xylose reductase (XR) and xylitol dehydrogenase (XDH), which convert xylose into xylitol and then to xylulose. XR (encoded by *XYL1*) requires NADH or NADPH, with a higher affinity for NADPH, while XDH (encoded by *XYL2*) is NAD-dependent (Fig. 1). Coexpression of both genes causes cofactor imbalance, leading to low xylose fermentation efficiency and xylitol accumulation (Krahulec et al. 2012). A genome-wide model of *S. cerevisiae* predicted that a balanced XR/XDH system increases ethanol production by 24.7% and reduces substrate utilization time by 70% (Ghosh et al. 2011). Computational design and site-specific mutagenesis of cofactor-binding domains improved cofactor specificity in XR and XDH, enhancing ethanol yield and reducing xylitol production in *S. cerevisiae* strains (Bengtsson et al. 2009, Lee et al. 2012). Coexpression of modified NADP-dependent XDH with native XR reduced xylitol yield and increased ethanol yield, with 32% increase xylose consumption rate (Matsushika and Sawayama 2008). The ratio of XR to XDH activity is crucial for improving xylose fermentation, with low *XYL2* expression contributing to xylitol accumulation (Kim et al. 2013a).

Xylulokinase (XK), an ATP-dependent enzyme that phosphorylates xylulose, catalyzes a key step in xylose catabolism and often limits ethanol production. Overexpression of XK in *S. cerevisiae* strains engineered with XR/XDH or XI pathways significantly reduces xylitol accumulation (Parachin et al. 2011). XK activity can be enhanced by derepressing the endogenous *XKS1* gene or expressing *XYL3* from *S. stipitis* (Jin et al. 2003). However, excessive XK activity may impair cell growth on xylose, likely due to ATP depletion caused by accelerated xylulose phosphorylation (Du et al. 2010).

Modifying genes in the pentose phosphate pathway (PPP) enhances ethanol production and reduces by-products like xylitol. Disruption of the *GRE3* gene, encoding a nonspecific aldose reductase responsible for xylitol production, improved ethanol yield to 0.347 g/g xylose and reduced xylitol accumulation by 85%, preventing xylitol inhibition of XI (Tanino et al. 2010, Romaní et al. 2015, Bamba et al. 2016). Disruption of the *PHO13* gene, which encodes an alkaline phosphatase involved in ATP balance during xylose metabolism, combined with *GRE3* knockout, enhanced cofermentation of glucose, cellobiose, and xylose, achieving 0.44 g/g ethanol yield and 48.6 g/l ethanol (Aeling et al. 2012). Targeting the oxidative branch of the PPP by deleting *ZWF1* (encoding glucose-6-phosphate dehydrogenase) and/or *GND1* (6-phosphogluconate dehydrogenase) while expressing *XYL1* and *XYL2* increased ethanol yield (Jeppsson et al. 2002, Verho et al. 2003). Combining overexpression of fungal NADP-dependent glyceraldehyde-3-phosphate dehydrogenase with *ZWF1* deletion further boosted ethanol production (Verho et al. 2003). Redox balance adjustments, including *GDH1* (NADPH-dependent glutamate dehydrogenase) deletion and *GDH2* (NADH-dependent glutamate dehydrogenase) overexpression, decreased xylitol, and increased ethanol yields (Roca et al. 2003). Overexpressing *noxE* from *Lactococcus lactis*, encoding a water-forming NADH-oxidase, reduced xylitol accumulation (Zhang et al. 2012). Deletion of *ALD6*, encoding NADP-dependent aldehyde dehydrogenase in the acetate biosynthetic pathway, improved xylose-to-ethanol conversion efficiency (Lee et al. 2012) (Fig. 1).

Engineered hexose transporters (Hxt) of *S. cerevisiae* were initially designed for xylose transport, but their instability under glucose-rich conditions due to glucose-induced degradation posed challenges (Farwick et al. 2014, Nijland et al. 2014, 2016). Substituting N-terminal lysines in Hxt1 and Hxt36 improved transporter stability and xylose fermentation under glucose-depleted conditions (Nijland et al. 2016). Moreover, the expression of a glucose-insensitive xylose transporter Mgt05196 (N360F) from *Meyerozyma guilliermondii* in a strain overexpressing XI, XK, and PPP genes, combined with *GRE3* and *PHO13* deletions, enhanced xylose fermentation and inhibitor tolerance (Li et al. 2016) (Fig. 1).

Adaptive evolution in xylose as the sole carbon source is necessary to develop an efficient *S. cerevisiae* strain for xylose utilization (Qi et al. 2015). Studies comparing the “omics” profiles of evolved strains with high xylose utilization and their parental strains with low capacity revealed that a key limiting factor for xylose fermentation is the lack of a signaling pathway in *S. cerevisiae* to recognize xylose and promote its utilization. Recent research showed that *S. cerevisiae* can sense xylose through glucose sensors Snf3 and Rgt2, which initiate the glucose signaling pathway (Wu et al. 2020). Deleting *RGT2* or *SNF3* reduced *HXT1* expression on xylose, with Snf3 being the primary sensor for high xylose concentrations. The *RGT1* gene encodes a transcriptional repressor that regulates the expression of *HXT* genes involved in hexose transport. Deleting *RGT1* derepresses these transporters, leading to increased uptake of xylose, which explains the observed increase in the specific xylose consumption rate. Additionally, deleting *PDE1* and *PDE2*, which encode cAMP phosphodiesterases, increased PKA activity, boosting xylose utilization and ethanol production. The *pde1Δ pde2Δ* mutant showed a 50% increase in xylose consumption and a 70% rise in ethanol production compared to the wild-type (Wu et al. 2020).

Attention has been paid to construction of *S. cerevisiae*, which grows and ferments third most abundant sugar of lignocellulosic hydrolysates, l-arabinose. Two different l-arabinose catabolizing pathways were identified in bacteria (Wisselink et al. 2007) and fungi (Richard et al. 2001, 2002). In the bacterial pathway, l-arabinose is isomerized to l-ribulose by arabinose isomerase (*araA*), then ribulose is phosphorylated to l-ribulose-5-phosphate by ribulokinase (*araB*), which in turn is converted to d-xylulose-5-phosphate by ribulose-5-phosphate-4-epimerase (*araD*) (Wisselink et al. 2007). Coexpression of *araA, araB*, and *araD* from *Lactobacillus plantarum* in *S. cerevisiae* led to arabinose fermentation (Wisselink et al. 2007). In fungi, l-arabinose is reduced to l-arabitol by aldose reductase, which is subsequently converted to l-xylulose by arabinitol dehydrogenase. l-xylulose is then reduced to xylitol by xylulose reductase and oxidized back to xylulose by XDH. The expression of the fungal arabinose utilization pathway (Gre3 aldose reductase from *S. cerevisiae*, arabinitol dehydrogenase and xylulose reductase from *Trichoderma reesei*, XDH from *S. stipitis*, and XK from *S. cerevisiae*) in *S. cerevisiae* resulted in low ethanol production with significant arabinitol accumulation, likely due to cofactor imbalances among the involved enzymes (Richard et al. 2001, 2002, Bettiga et al. 2009). The bacterial redox-independent l-arabinose utilization pathway in *S. cerevisiae* presents a more promising strategy (Zhang et al. 2015a) (Fig. 1).

The yeast *S. cerevisiae* efficiently ferments the hexoses glucose, fructose, mannose, and galactose, though galactose fermentation is less efficient due to glucose-mediated repression. In contrast, mannose uses the glucose transport system, enabling cofermentation with glucose (Madhavan et al. 2012). The combined deletion of the genes *GAL6, GAL80*, and *MIG1* involved in negative regulation of galactose catabolism resulted in a partial coconsumption of glucose and galactose in aerobic conditions (Ostergaard et al. 2001). Normally, galactose metabolism in *S. cerevisiae* requires respiration. However, a cox9Δ gal80Δ double mutant was identified that could ferment galactose efficiently under anaerobic conditions (Quarterman et al. 2016). Interestingly, a natural strain of *S. cerevisiae* (NRRL Y-1528) has been described that catabolizes galactose more efficiently than glucose or mannose. This strain exhibits simultaneous fermentation of all hexoses present in hydrolyzed biomass, suggesting a possible defect in carbon catabolic repression. Such a defect may alleviate glucose-mediated repression of galactose utilization, enabling more efficient sugar cofermentation (Keating et al. 2004) (Table 1).

**Table 1.** Ethanol production by yeast species.

Strain	Carbon source	Ethanol production (g/l)	Temperature (^o^C)	References
*S. cerevisiae*				
SR8	Xylose	40.0	30	Shin et al. (2019)
NRRL Y-1528	Steam-exploded Douglas-fir	15.0	30	Keating et al. (2004)
KCCM 1129	Kariba weed hydrolysate	14.0	30	Kityo et al. (2020)
80000012a	Corn stover	59.8	39	Liu et al. (2014)
*S. (Pichia) stipitis*				
NRRL Y-7124	Glucose/Xylose	17.2	30	Nakanishi et al. (2017)
CBS 6054	Xylose	32.4	30	Shin et al. (2019)
KCTC 17 574	Kariba weed hydrolysate	16.0	30	Kityo et al. (2020)
*K. marxianus*				
YZJ088	Xylose	20.3	45	Zhang et al. (2015b)
IMB3	Barley straw	12.0	45	Boyle et al. (1997)
CECT 10 875	Paper sludge	17.7	42	Ballesteros et al. (2002)
DBTIOC-35	Sugarcane bagasse hydrolysate	16.1	45	Saini et al. (2015)
CBS6556	Poplar	49.0	43	Sengupta et al. (2022)
MSS6.3	Rice straw	10.9	45	Avchar et al. (2022)
*O. (Hansenula) polymorpha*				
BEP/cat8Δ/*DAS1*/*TAL2*	Xylose	16.5	45	Kurylenko et al. (2018)
A107	Xylose	20.9	45	Vasylyshyn et al. (2025, unpublished)

Wild-type *S. cerevisiae* strains cannot ferment intermediate sugars like cellobiose and cellotriose from cellulose hydrolysis (Turner et al. 2018). To address this, cellobiose-metabolizing *S. cerevisiae* strains have been developed by introducing a cellobiose metabolic pathway, utilizing either intracellular β-glucosidase (GH1-1) or cellobiose phosphorylase (CBP), along with an energy-consuming transporter (CDT-1) or a passive facilitator (CDT-2) (Choi et al. 2022). Mutant CDTs, such as CDT-1 (F213L) and CDT-2 (N306I), have significantly improved cellobiose transport, enhancing fermentation performance (Ha et al. 2013, Kim et al. 2018). Another strategy involves heterologous cellulase expression. For instance, expressing a cellulase gene from *Ampullaria gigas* in *S. cerevisiae* led to a 23-fold increase in endo-1,4-β-glucanase activity and a 37.7-fold higher ethanol yield (Yang et al. 2018). Additionally, disrupting the *CWP2* and *YGP1* genes in *S. cerevisiae* INVSc1 increased β-glucosidase activity and ethanol production from cellobiose, with the *YGP1* disruptant strain producing 59% more ethanol than the wild-type strain (Arnthong et al. 2022).

Fermentation of lignocellulose-derived hydrolysates is frequently hindered by the presence of inhibitory compounds formed during biomass pretreatment, including furfural, hydroxymethylfurfural (HMF), weak organic acids, and phenolic compounds (Palmqvist and Hahn-Hägerdal 2001). To mitigate these inhibitory effects, *S. cerevisiae* strains with enhanced ethanol production capacity in the presence of such compounds have been developed via directed evolution and ALE strategies (Hawkins et al. 2013, Parreiras et al. 2014). In addition, molecular engineering approaches have been employed. For instance, detoxification of furfural and HMF through their conversion to furfuryl alcohol and furan dimethanol, respectively, has been achieved by overexpressing endogenous oxidoreductases in *S. cerevisiae*, such as alcohol dehydrogenases (*ADH1, ADH6*, and *ADH7*) (Petersson et al. 2006, Almeida et al. 2008, Liu et al. 2008) and the aldo-keto reductase (*GRE2*) (Moon and Liu 2012). Gorsich et al. (2006) demonstrated that resistance to furfural stress correlates with enhanced expression of genes in the PPP, including *ZWF1, GND1, RPE1*, and *TKL1*. Furthermore, overexpression of the *PAD1* gene, encoding phenylacrylic acid decarboxylase, has been linked to improved growth and ethanol production in dilute-acid hydrolysates (Larsson et al. 2001). An additional strategy for enhancing tolerance to both fermentation inhibitors and ethanol involves modulation of intracellular spermidine levels in *S. cerevisiae* (Kim et al. 2015). Intracellular spermidine contents increases due to double deletion of *OAZ1* (ornithine decarboxylase antizyme) and *TPO1* (polyamine transport protein) genes, along with and the overexpression of *SPE3* (spermidine synthase) (Kim et al. 2015). In a later study, Liu and Ma (2020) also investigated the transcriptomic responses of evolved strains tolerant to furfural and HMF to the influence of these inhibitors. The results of comparative transcriptomic analysis identified the cell wall response, endogenous and exogenous detoxification pathways, and specific transcription factors such as Yap1, Met4, Msn2/4, and Pdr1/3 as key components necessary for inhibitor tolerance.

## Non-*Saccharomyces* yeasts: *S. (Pichia) stipitis, K. marxianus*, and *O. (Hansenula) polymorpha*

Nonconventional yeasts may have advantages in fermentation processes, such as tolerance to high temperatures and inhibitors. However, unlike *Saccharomyces*, these organisms have been less studied, and they represent important areas that should be further explored (Radecka et al. 2015).

*Scheffersomyces (Pichia) stipitis* is considered a competitive candidate for cellulosic ethanol production due to its ability to ferment a broad range of sugars present in saccharified lignocellulose, including glucose, xylose, mannose, galactose, and cellobiose (Jeffries et al. 2007, Bader et al. 2021). Additionally, the yeast has been genetically engineered to achieve high yields of lactic acid and xylitol (Lee et al. 1986, Targonski 1992, Kim et al. 2001, Conceicao et al. 2003, Ilmen et al. 2007). Fermentation studies with lignocellulosic hydrolysates have demonstrated sugar conversion yields approaching 80% of the theoretical maximum (Nigam 2001, Santos et al. 2016, Bader et al. 2021). However, its practical application is limited by relatively low fermentation rates, poor ethanol tolerance, and an inability to grow under strictly anaerobic conditions (du Preez et al. 1989, Grootjen et al. 1990, Shi and Jeffries 1998). Additionally, due to its dependence on oxygen, *S. stipitis* often reconsumes the ethanol produced, leaving a substantial amount of xylose unfermented in the medium (Harner et al. 2015).

Initial metabolism of xylose in *S. stipitis* is similar to other natural xylose-fermenting yeasts (Fig. 1). Xylose is first reduced by XR (aldose reductase, gene *XYL1*) to xylitol (Verduyn et al. 1985). This enzyme can utilize both NADH and NADPH as cofactors but shows a higher affinity for NADPH. Xylitol is then oxidized to xylulose by XDH (gene *XYL2*), which is strictly NAD-dependent (Metzger and Hollenberg 1995), maintaining cofactor balance. This efficient redox balance minimizes xylitol accumulation and enhances ethanol yield (Skoog and Chandra 1990, Kurtzman and Suzuki 2010, Balagurunathan et al. 2012). The final step of xylose metabolism involves XK (gene *XYL3*), which phosphorylates xylulose to xylulose-5-phosphate, an intermediate of the PPP (Jin et al. 2002). Mechanisms of maintaining efficient redox balance and absence of xylitol accumulation in *S. stipitis* should be further elucidated. This phenomenon is likely associated with an auxiliary pathway involving four enzymes: *GDH2* (NAD-dependent glutamate dehydrogenase), which catalyzes the conversion of 2-oxoglutarate to l-glutamate using NADH; *GAD2* (glutamate decarboxylase), which forms GABA (i.e. gamma-aminobutyric acid) via l-glutamate decarboxylation; *UGA1* (4-aminobutyrate aminotransferase), which converts GABA to succinate semialdehyde; and *UGA2* (succinate semialdehyde dehydrogenase), which oxidizes succinate semialdehyde to succinate using NADP. This pathway contributes to NADPH regeneration and redox homeostasis. Deletion of *XYL1* or *XYL2* disrupts xylose metabolism, causing xylitol accumulation, while *XYL3* overexpression enhances xylose utilization. Alterations in *UGA1* and *UGA2* impact NADPH regeneration, affecting ethanol yield and growth under oxygen-limited conditions (Jeffries et al. 2007, Jeffries and Van Vleet 2009).

Sugar uptake is a key rate-limiting step in ethanol production by *S. stipitis* (Agbogbo et al. 2006). A high-affinity transport system specific for xylose has been identified in this yeast (Hahn-Hägerdal et al. 2001). The genes *SUT1, SUT2*, and *SUT3* encode glucose transporters in *S. stipitis* (Weierstall et al. 1999). Sut2 and Sut3 share high similarity with glucose transporters from *S. cerevisiae* and show a stronger affinity for glucose than for xylose. Expression of *SUT1* occurs independently of oxygen, while *SUT2* and *SUT3* are expressed only under aerobic conditions and are not regulated by the carbon source. Deletion of *SUT1* eliminates low-affinity xylose transport in *S. stipitis* (Weierstall et al. 1999).

An important point in the production of 2 G ethanol is that the strain used in the process must tolerate the inhibitors present in the hydrolysates, avoiding the need for detoxification and reducing costs. Nakanishi et al. (2017) investigated the stress tolerance of *S. stipitis* NRRL Y-7124 cultivated in enzymatic hydrolysates derived from alkaline-pretreated sugarcane bagasse. The study employed a combination of cell recycling and fed-batch fermentation under oxygen-limited conditions. This approach, which is a form of adaptive laboratory evolution (ALE), allowed the strain NRRL Y-7124 to produce 17.2 g/l ethanol with a yield of 0.285 g ethanol/g sugar in a medium containing 43 g/l glucose and 15 g/l xylose after four cycles (Table 1). Xylose consumption increased by 50% from the first to the fourth cycle, demonstrating that cell recycling can help overcome glucose inhibition when both xylose and glucose are present. In addition to ALE, other strategies to improve stress tolerance in *S. stipitis* include random mutagenesis (Bajwa et al. 2009) and genome shuffling (Zhang and Geng 2012) via protoplast fusion of genetically diverse strains. UV mutagenesis has also been used to select ethanol-tolerant strains, leading to improved ethanol yields (Watanabe et al. 2011). Random mutagenesis and screening for resistance to 3-bromopyruvate allowed the isolation of insertion mutants with altered ethanol production from glucose and xylose. The insertion occurred in a homolog of *S. cerevisiae HEM25* (*YDL119C*), a mitochondrial glycine transporter involved in heme synthesis. The observed phenotype may result from defects in respiration caused by *HEM25* disruption enhancing consequently fermentation efficiency (Berezka et al. 2021).

Additional experimental studies were conducted to evaluate whether inhibiting the respiratory chain enhances ethanol production in *S. stipitis* (Acevedo et al. 2017). Inhibitors targeting the respiratory chain were introduced into batch cultures of *S. stipitis* utilizing xylose under conditions of limited oxygen supply. Specifically, salicylhydroxamic acid (SHAM) was used to inhibit alternative oxidase, while potassium cyanide and sodium azide were employed to inhibit cytochrome oxidase (COX). The inhibitors resulted in a decrease in ethanol yield, with SHAM causing a reduction of over 25%, and potassium cyanide and sodium azide leading to a reduction of more than 50%. Given that all previously identified *in silico* deletions were related to the respiratory chain, the inhibition of the NADH dehydrogenase complex I was also investigated. The addition of rotenone, which inhibits Complex I, increased the ethanol yield by up to 13% (Acevedo et al. 2017). The combination of SHAM and antimycin A (AA) abolished respiration, depleting the glycolytic flux using both carbon sources tested, leading to increased ethanol production of 21.05 g/l for glucose, and 8.3 g/l ethanol using xylose. In contrast, inhibition of only the AA-sensitive respiration, caused increased ethanol production to 30 g/l using glucose, and 11.3 g/l from xylose (Granados-Arvizu et al. 2021).

Another yeast of interest is *K. marxianus* due to its combination of beneficial traits, such as the fastest known eukaryotic growth rate, high thermotolerance (strains can grow at 47°C, 49°C, and even up to 52°C) (Hughes et al. 1984, Banat et al. 1992, Nonklang et al. 2008, da Silva et al. 2018), and the ability to produce ethanol at temperatures above 40°C (Madeira-Jr and Gombert 2018). Moreover, it can metabolize a wide range of carbon sources, including pentoses (e.g. xylose, l-arabinose), hexoses (e.g. mannose, galactose, glucose, and maltose), disaccharides (e.g. cellobiose), and various low-cost substrates such as sugar syrup molasses, hemicellulose hydrolysates, corn silage juice, and dairy industry by-products (Duan et al. 2019, Zhou et al. 2018, Hua et al. 2019, Mo et al. 2019, Yu et al. 2021, Lyu et al. 2021, Hang et al. 2003, Ozmihci and Kargi 2007, Martínez et al. 2017). Given this broad substrate spectrum and robust performance under industrial conditions, *K. marxianus* is increasingly recognized as a promising alternative to *S. cerevisiae* for biotechnological applications.

Through genetic engineering (disruption of the *KmXYL1* gene and expression of the *XYLA* gene of XI from *Orpinomyces sp*. it was possible to construct recombinant strains of *K. marxianus* with increased capacity to produce ethanol through direct xylose assimilation via XI (Wang et al. 2013) or a combination of a NADPH-preferring XR (overexpression of *NcXYL1* gene from *Neurospora crassa*) and NADPH-preferring XDH (*XYL2* gene from *S. stipitis*) (Zhang et al. 2013, 2015b). To decrease the level of byproducts, such as glycerol and acetic acid, disruption of *GPD1* coding for glycerol-3-phosphate dehydrogenase was done. In this study, the highest ethanol productivity from xylose was obtained as compared to other works. The resulting engineered *K. marxianus* YZJ088 strain produced 44.95 g/l ethanol from 118.39 g/l xylose with a productivity of 2.49 g/l/h and yield 0.38 g/g at 42°C. However, this strain accumulated large amounts of xylitol (11 g/l) and xylulose (near 10 g/l). At 45°C, ethanol accumulation dropped more that two folds: from 44.95 to 20.27 g/l (Zhang et al. 2015b) (Table 1). Adaptive evolution was also shown to enhance ethanol production and tolerance to xylose (Kwon et al. 2019) and glucose (Mo et al.^2019^ ). This was achieved through mutations in the *KmXYL1* and *KmXYL2* genes, as well as upregulation of genes (*PPT2, FAD2*, and *TAZ1*) involved in unsaturated fatty acid biosynthesis and downregulation of genes (*ACC1, TSC13*, and *FAS1*) related to low ethanol tolerance (Mo et al. 2019, Silva et al. 2015, Kwon et al. 2019). evaluated the fermentative potential of *K. marxianus* ATCC 36907 during the cultivation in sugarcane bagasse cellulosic and hemicellulosic hydrolysates and in semidefined medium with a similar sugar composition. According to these authors, glucose assimilation resulted in the formation of ethanol, while xylose assimilation resulted in xylitol production as a main product and ethanol as a by-product. Ethanol productivity (0.86 g/l/h) was 10-fold higher in the semidefined-medium than the cellulosic hydrolysate. However, the yeast performance was much better in the hemicellulosic hydrolysate than in the semidefined medium, demonstrating that the toxic compounds did not inhibit sugar metabolism in this yeast strain. Moreover, the *K. marxianus* strains BUNL-21 and DMKU3-1042 were capable of producing ethanol from xylose in the presence of 10 mM HMF or furfural, with production levels nearly identical to those observed in their absence (Nitiyon et al. 2016, Zhang 2019).

Fermentative behavior of *K. marxianus* may vary according to the strain used due to its high metabolic diversity and high degree of intraspecific polymorphism (Lane et al. 2011). Several studies have demonstrated the potential of *K. marxianus* for 2 G ethanol production, with ethanol titers ranging from 7.35 to 66.2 g/l when using SSF and different biomasses, including lignocellulosic residues, agro-industrial by-products, and paper sludge (Kim et al. 2013b, Yu et al. 2013, Camargo et al. 2014, Yan et al. 2015, da Costa et al. 2015, Saini et al. 2015). For instance, with thermotolerant *K. marxianus* IMB strains (IMB1, IMB2, IMB3, IMB4, and IMB5) (Banat et al. 1992), the ability to produce ethanol under SSF at 45°C was verified, with maximum theoretical yields between 67% and 80% (Faga et al. 2010). IMB3 strain achieved a maximum concentration of 12 g/l ethanol (maximum theoretical yield 98%) at 45°C from the 40% cellulose present in pretreated barley straw with sodium hydroxide (Boyle et al. 1997). Another strain studied, *K. marxianus* CECT 10875, reached an ethanol concentration of 17.7 g/l, with a maximum theoretical yield of 97.7%, from paper sludge at 42°C (Ballesteros et al. 2002). SSF with *K. marxianus* DBTIOC-35 at 42°C and 45°C using 10% biomass loading resulted in ethanol titers of 29.0 g/l and 16.1 g/l, respectively. By increasing the biomass loading to 20%, SSF without presaccharification (PSSF) led to an increased ethanol production (66.2 g/l with 83.3% yield), which was superior to SSF with PSSF. This represents an advantage in terms of reducing the overall biomass-to-bioethanol process time and enhancing bioethanol concentration with high yields and productivities (Saini et al. 2015). With *S. cerevisiae*, a slightly lower ethanol concentration, 62 g/l, was found when a high solid load of 26% was employed in SSF (Paschos et al. 2015). To further expand the substrate range, Anandharaj et al. (2020) engineered *K. marxianus* to express the largest known cellulosome complex on its cell surface, displaying 63 enzymes that enabled this yeast to efficiently degrade cellulosic substrates. As a result, ethanol accumulation reached 3.09 g/l from avicel and 8.61 g/l from phosphoric acid-swollen cellulose (Anandharaj et al. 2020). Avchar et al. (2022) isolated 92 thermo-tolerant yeast strains from distillery effluent and molasses samples. In the isolated strains, nine species were contained such as *S. cerevisiae, O. polymorpha*, and *Candida ethanolica*. Among the strains, a *K. marxianus* strain MSS6.3 produced maximum concentration of ethanol at 45°C (Table 1).

*Ogataea (Hansenula) polymorpha* is one of the most thermotolerant species of yeast known. This yeast has the ability to grow up to a temperature of 50°C and higher (Ishchuk et al. 2009, Sibirny et al. 2025 in “Extremophilic Yeasts” ed. by P. Buzzini. Springer, in press). Wild-type strains of this yeast are able to ferment glucose, xylose, mannose, maltose, and cellobiose into ethanol, but are not able to ferment galactose and l-arabinose (Ryabova et al. 2003, Radecka et al. 2015). Sugar fermentation in this yeast is most efficient under conditions of limited aeration and even at 45°C–48°C it is still sufficiently vigorous (Ishchuk et al. 2009). Riboflavin deficiency activates alcoholic fermentation, apparently, due to inhibiting respiration (Ryabova et al. 2003).

To overcome cofactor imbalance during xylose fermentation in *O. polymorpha*, two approaches were applied. First, XR and XDH were replaced with bacterial XI (*xylA* from *E. coli* or *S. coelicolor*), enabling cofactor-independent xylose conversion. However, ethanol production remained low (0.15–0.6 g/l) (Dmytruk et al. 2008a, Voronovsky et al. 2005). Second, XR was engineered to reduce NADPH affinity via site-directed mutagenesis, resulting in a 17-fold decrease in NADPH dependence while maintaining NADH activity. The strain expressing modified XR, native XDH, and XK showed reduced xylitol formation and a 2-fold increase in ethanol yield (1.3 g/l) (Dmytruk et al. 2008b). To achieve higher ethanol production from xylose, several successful metabolic engineering approaches were combined to modify the genome of the 2EthOH^−^ mutant, which is unable to utilize ethanol as a sole carbon source. Further overexpression of the genes *XYL1m, XYL2*, and *XYL3*, encoding a modified XR, native XDH and XK, respectively, on the background of nonidentified mutation in the strain 2EthOH^−^, led to a substantial increase in ethanol accumulation during xylose fermentation (7.4 g/l at 45°C relative to 0.6 g/l in the wild-type strain NCYC495) (Kurylenko et al. 2014).

Xylose is fermented to ethanol primarily via the PPP, while gluconeogenesis can compete with fermentation. The *CAT8* gene encodes a zinc-finger cluster protein regulating genes involved in gluconeogenesis, ethanol utilization, the glyoxylate cycle, and the diauxic shift from fermentation to respiration (Haurie et al. 2001). Deletion of *CAT8* in *O. polymorpha* significantly increased ethanol production from xylose, impairing cell respiration and utilization of ethanol and glycerol, suggesting its role in gluconeogenesis (Ruchala et al. 2017). Overexpression of *CAT8* had the opposite effect, reducing ethanol production. The *cat8Δ* mutant from an advanced ethanol producer accumulated 30% more ethanol than the parental strain, reaching 12.5 g/l at 45°C, the highest titer for high-temperature xylose fermentation (Ruchala et al. 2017). Transcription activator Hap4 is a key regulator of respiratory activity in *S. cerevisiae* (Olesen and Guarente 1990). Two functional homologs, Hap4-A and Hap4-B, were identified in *O. polymorpha* (Sybirna et al. 2010). Hap4-A is crucial for xylose metabolism, with its deletion enhancing and overexpression reducing ethanol production from xylose (Kurylenko et al. 2021). Tup1, a global repressor, regulates genes involved in carbon metabolism (Courey and Jia 2001). Deletion of *TUP1* activates xylose fermentation but decreases glucose fermentation, while high *TUP1* expression inhibits both (Kurylenko et al. 2021). Azf1, a homolog of the *S. cerevisiae* transcription factor, regulates carbon source utilization and glucose sensing. In *O. polymorpha*, deletion of *AZF1* impairs both glucose and xylose fermentation, while its overexpression increases ethanol production, especially from xylose (Semkiv et al. 2022).

**Figure 2.**
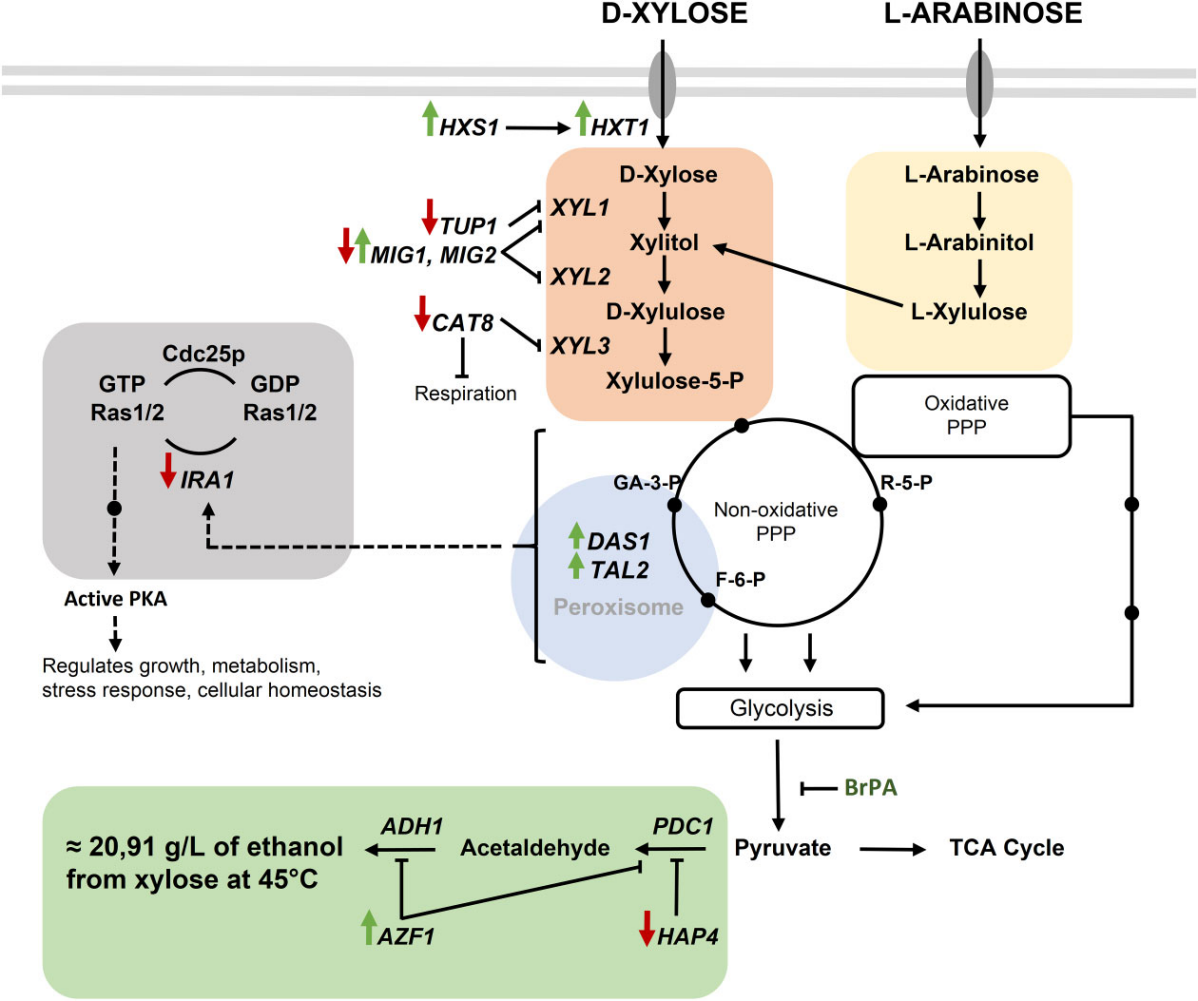
Genes involved in the regulation of pentose sugars alcoholic fermentation in *O. polymorpha* yeast. Enzymes and transporters include *XYL1* (xylose reductase), *XYL2* (xylitol dehydrogenase), *XYL3* (xylulokinase), *ADH1* (alcohol dehydrogenase), *PDC1* (pyruvate decarboxylase), *HXT1* (low-affinity hexose transporter), and *HXS1* (hexose-sensing receptor). Transcriptional regulators include *CAT8* and *AZF1* (transcription activators), *MIG1, MIG2, TUP1*, and *HAP4* (transcription factors). *DAS1* (dihydroxyacetone synthase) and *TAL2* (peroxisomal transketolase and transaldolase, respectively) play roles in pentose metabolism. The diagram also represents signal transduction pathways related to *IRA1* deletion. Arrows indicate decreased and increased gene expression.

Earlier, hexose transporter-like sensor gene *HXS1* in *O. polymorpha* was described (Stasyk et al. 2008). Recent studies demonstrated that *HXS1* is crucial for both glucose and xylose metabolism, as well as their alcoholic fermentation: deletion of *HXS1* suppressed, while its overexpression stimulated these processes (Semkiv et al. 2022). To improve xylose utilization and cofermentation with glucose, the native hexose transporter *HXT1* was engineered to prevent glucose inhibition and endocytosis, which enabled simultaneous consumption of glucose and xylose and led to increased ethanol production from their mixture (70% glucose, 30% xylose). In addition, heterologous transporters *HXT7* and *GAL2* from *S. cerevisiae*, modified to improve sugar transport, were overexpressed in *O. polymorpha* BEP/cat8Δ strain, although their effects on sugar uptake were limited, with *GAL2* showing moderate and *HXT7* minimal positive impacts (Vasylyshyn et al. 2020). Further increase in ethanol production from xylose was achieved by selecting mutants capable of growing on l-arabinose as the sole carbon source (Vasylyshyn et al. 2025, unpublished). These strains demonstrated significantly enhanced ethanol production from xylose (up to 20 g/l) (Table 1) and minor ethanol formation from l-arabinose (∼0.2 g/l) at 45°C. Whole-genome sequencing of the selected mutants revealed two key mutations: one in a putative arabinose-5-phosphate isomerase gene, potentially involved in l-arabinose metabolism, and another in a Ras-GTPase activating protein homologous to *S. cerevisiae* Ira1/Ira2, which are negative regulators of the Ras-cAMP signaling pathway. Deletion of *IRA1* in *O. polymorpha* conferred advantages under aerobic fermentation of pentose sugars and resistance to high xylose concentrations (Vasylyshyn et al. 2025, unpublished work) (Fig. 2).

Peroxisomal transaldolase Das1 and transketolase Tal2 are involved in xylose alcoholic fermentation in *O. polymorpha* (Kurylenko et al. 2018). To clarify their role in ethanol production, the corresponding genes *DAS1* and *TAL2* were overexpressed in the *O. polymorpha* NCYC495 strain. *TAL2* overexpression increased ethanol production 1.5-fold, while *DAS1* overexpression had a stronger effect, raising ethanol levels 2.3-fold compared to the parental strain. Cooverexpression of *DAS1* and *TAL2* in a high-performing xylose-fermenting strain increased ethanol accumulation to 16.5 g/l at 45°C—30–40 times more than in the wild type. Although peroxisomal transketolase and transaldolase are dispensable for growth on xylose as a sole carbon source (unlike their cytosolic counterparts *TKL1* and *TAL1*), they are essential for efficient xylose-to-ethanol conversion. In contrast, deletion of *TKL1* and *TAL1* abolished growth on xylose but only partially suppressed its fermentation. Moreover, overexpression of *TKL1* and *TAL1* also enhanced ethanol production from xylose (Kurylenko et al. 2018) (Table 1).

Wild-type *O. polymorpha* NCYC495 cannot assimilate cellobiose. To overcome this, cellobiose-fermenting strains were developed from the improved xylose-fermenting strain BEP/cat8Δ by introducing heterologous genes: *gh1-1* (β-glucosidase), *CDT-1 m* and *CDT-2 m* (cellodextrin transporters from *N. crassa*), and *CBP* (CBP from *Saccharophagus degradans*). Further metabolic engineering, adaptive evolution, and mutagenesis yielded mutants with enhanced high-temperature fermentation of cellobiose and efficient coutilization of major sugars in lignocellulosic hydrolysates (Vasylyshyn et al. 2024). Today, a number of microorganisms are also known to possess a natural or acquired ability to ferment cellobiose into ethanol. For example, *S. cerevisiae*, which accumulates 38 g/l of ethanol at 30°C (Choi et al. 2022), *Myceliophthora thermophile–*11.3 g/l of ethanol at 45°C–50°C, (Li et al. 2020 ), *Zymobacter palmae*—10 g/l of ethanol at 30°C, (Yanase et al. 2005). The *O. polymorpha* currently produces a maximum of only 5 g/l of ethanol at 45°C (Vasylyshyn et al. 2024). Thus, the ethanol yield in the best obtained *O. polymorpha* advanced ethanol producer from cellobiose obtained by metabolic engineering and classical selection approaches is not high enough for economic feasibility (Fig. 2).

Based on published data, the ethanol yield of the constructed *O. polymorpha* recombinant strain (0.39 g/g xylose) (Kurylenko et al. 2018) is close to that described for *S. stipitis* (0.35–0.44 g/g xylose) (Jeffries et al. 2007) and *Spathaspora passalidarum* (0.42 g/g xylose) (Long et al. 2012). However, this yield was achieved for *O. polymorpha* at 45°C, whereas the compared organisms are mesophilic and therefore unable to grow and ferment at such a high temperature. Among the thermotolerant ethanol-producing strains, the most promising one is an engineered *K. marxianus* strain with an ethanol yield of 0.38 g/g xylose at 42°C, but a lower yield at 45°C (0.27 g/g xylose). An additional advantage of the *O. polymorpha* recombinant strain, in contrast to the recombinant *K. marxianus*, is that no xylitol accumulation was observed. Further improvement of xylose fermentation in *O. polymorpha* may depend on optimizing pentose uptake and enhancing resistance to inhibitors generated during lignocellulosic biomass pretreatment. However, its response to aldehydes, phenolic compounds, and weak acids present in acid hydrolysates remains insufficiently characterized.

## *Saccharomyces cerevisiae* or nonconventional yeasts? Outlook

As outlined above, the past decade has seen a surge in research focused on genetic engineering, metabolic rewiring, flux analysis, and adaptive evolution of *S. cerevisiae* and nonconventional yeasts, aiming to reduce production costs and enhance bioethanol generation from lignocellulosic hydrolysates. Moreover, engineered strains capable of efficient sugar uptake and simultaneous fermentation of mixed substrates are key to achieving high ethanol titers, yields, and productivity. However, many problems still remain, such as the presence of toxic compounds in lignocellulosic hydrolysates at high concentrations, which severely impair yeast’s ability to efficiently utilize sugars, especially xylose (Wei et al. 2021, Ding et al. 2023). Moreover, the strong glucose repression effect further limits the simultaneous consumption of mixed sugars, reducing the overall efficiency of ethanol production (Caspeta et al. 2015).

*Saccharomyces cerevisiae* strains constructed for production of 2 G ethanol efficiently ferment, in addition to glucose, abundant pentose sugars of lignocellulosic hydrolyzates, xylose, and l-arabinose. Strains fermenting galactose have also been isolated. There are also known strains, which could ferment different sugars of hydrolyzates simultaneously due to elaboration of specific xylose transporters and expression of genes responsible for xylose catabolism under control of strong constitutive promoters. There are strains resistant to inhibitors found in lignocellulosic hydrolysates, which have paved the way for pilot-scale production of 2 G ethanol using engineered *S. cerevisiae* strains. However, if the hydrolysate is highly toxic, the growth and metabolism of strains with lower robustness are significantly inhibited when glucose is used as a carbon source (Sun et al. 2024). Therefore, enhancing the tolerance of industrial strains to toxic components of lignocellulosic hydrolysates remains a top priority in the field. Through genome-wide knockout library screening and bioinformatic analysis, identified a set of genes for further modification of *S. cerevisiae* to enhance its tolerance, representing a significant contribution to future improving the efficiency of lignocellulosic bioethanol fermentation (Li et al. 2023). Additionally, although significant progress has been made in engineering *Saccharomyces* strains for improved sugar utilization and thermotolerance (Favaro et al. 2013, Pandey et al. 2019, Prado et al. 2020), to date, no strains have been developed that can efficiently ferment major hydrolysate sugars with high ethanol yield under elevated temperatures typical of SSF processes. Whole genome sequencing of recently isolated thermotolerant *S. cerevisiae* strain AH465 with biotechnological significance was done. It is believed that obtained genomic information will give a useful insight into the elucidation of thermotolerant mechanism in yeast and lead to molecular breeding of yeast strains for industrial biofuel production (Sasano et al. 2024).

The Brazilian bioethanol sector provides a prominent example of the industrial deployment of recombinant *S. cerevisiae* strains, with up to 30% of its fuel ethanol derived from sugarcane bagasse (Rossi et al. 2021). Nevertheless, large-scale deployment of 2 G bioethanol technologies has encountered considerable challenges. Major producers like Granbio and Raízen identified biomass pretreatment as a key bottleneck, largely due to reliance on imported technologies that required rapid adaptation to local feedstocks such as bagasse and straw (Furtado et al. 2020). In addition, companies like Petrobras, Odebrecht Agroindustrial, and Abengoa ultimately discontinued their 2 G ethanol initiatives. Further constraints, such as limited microbial growth or ethanol tolerance when real lignocellulosic hydrolysates are used, have also been reported (Susmozas et al. 2020). These hurdles highlight the ongoing need for development of robust, genetically optimized yeast strains capable of withstanding the harsh conditions of industrial-scale 2 G ethanol production (Table 2).

**Table 2.** Advantages and limitations of engineered *S. cerevisiae* versus native pentose-fermenting yeasts in the bioconversion of lignocellulosic sugars to ethanol.

Feature	*S. cerevisiae* (engineered)	*S. stipitis*	*K. marxianus*	*O. polymorpha*
Xylose fermentation	Requires genetic engineering (XR/XDH, XI, etc.)	Natural xylose fermenter	Engineered strains show improved performance	Engineered strains show improved performance
Ethanol tolerance	High	Low	Moderate	Moderate
Temperature tolerance	Moderate (∼30°C–39°C)	Low–moderate	High (up to 52°C)	High (up to 50°C)
Substrate range	Glucose, galactose, mannose, cellobiose	Glucose, xylose, mannose, galactose, cellobiose	Very broad (mono-, di-, some polysaccharides)	Glucose, xylose, mannose, cellobiose
Cofermentation with glucose	Engineered strains allow coconsumption	Glucose repression observed	Variable across strains	Advanced strains coferment at 45°C
Tolerance to inhibitors	High in evolved/engineered strains	Low (can be improved via ALE, mutagenesis)	Variable; some strains tolerate HMF, furfural	Uncharacterized
Fermentation under anaerobic conditions	Yes (native)	No (requires oxygen or microaerobic)	No (requires oxygen or microaerobic)	No (limited aeration)
Industrial maturity	High (industrial use, pilot-scale)	Moderate (prepilot)	Moderate (nonlignocellulosic industry use)	Low
Byproduct accumulation	Xylitol, acetate (if poorly engineered)	Minimal due to redox balance	High xylitol/xylulose in some strains	Minimal in engineered strains
Best ethanol titer reported (xylose)	∼40 g/l at 30°C (engineered)	∼32.4 g/l at 30°C	∼45 g/l at 42°C	∼21 g/l at 45°C

Research on nonconventional yeasts remains relatively limited, particularly regarding their genetic characteristics and metabolic pathways. However, native strains of non-*Saccharomyces* yeasts demonstrate notable potential due to their ability to utilize a broad range of sugars. It has been suggested that certain metabolic constraints, such as inefficient substrate utilization and limited ethanol production, can be overcome through strategies like metabolic engineering, ALE, and random mutagenesis (Kręgiel et al. 2017). Targeted efforts to modulate the NADH pool or to adjust the NADH/NADPH ratio of enzymes within the xylose oxidoreductase system may also contribute to improved fermentation performance. Likewise, heterologous XIs could be expressed in yeast, such as *S. stipitis* and *K. marxianus*, which have shown good xylose transport, appropriate downstream gene regulation, and low xylitol accumulation (Sharma et al. 2018). Further advancements could focus on the identification and engineering of pentose transporters from non-*Saccharomyces* species that are not inhibited by glucose and facilitate efficient xylose uptake. Improved thermotolerance and resistance to inhibitory compounds would also be essential traits for these yeasts under industrial fermentation conditions.

Similarly, improvements have been made in *S. stipitis* strains for xylose fermentation on lignocellulosic hydrolysates, addressing issues such as glucose catabolite repression and inhibitor resistance. Despite these advantages, challenges persist*. Scheffersomyces stipitis* strains typically have lower ethanol tolerance compared to *S. cerevisiae*, which can limit their efficiency in industrial-scale fermentations. In addition, most nonconventional yeasts require oxygen for growth, adding complexity to fermentation processes and potentially necessitating specialized bioreactor designs (Bonan et al. 2019, Nosrati-Ghods et al. 2020). Plans are underway to establish pilot plants for 2 G ethanol production based on *S. stipitis* strains, aiming to leverage its unique metabolic capabilities for commercial applications (Table 2).

Although several potential features of *K. marxianus* as a bioethanol producer, such as thermotolerance (Lertwattanasakul et al. 2015, Madeira-Jr and Gombert 2018) and the capacity to assimilate different carbon sources (Parrondo et al. 2009, Sofia et al. 2013, Magalh-aes-Guedes et al. 2013), have already been described, its application for 2 G cellulosic ethanol production from lignocellulosic biomass has not yet been reported at the pilot or industrial scale. Nevertheless, successful implementations for ethanol production from other substrates, such as starch and lactose, have been documented. In particular, processes employing *K. marxianus* for ethanol fermentation from cassava starch hydrolysates and sugar cane syrup have been developed in Thailand (Pimpakan et al. 2012, Waraporn et al. 2013, Rugthaworn et al. 2014). There are still some problems for the use of *K. marxianus* strains in industrial environments, since some of them present greater accumulation of xylitol instead of ethanol, their low viability at temperatures above 40°C, and the scarce information on their genetics and metabolism (de Barros et al. 2017). To date, only a limited number of studies have compared the performance of *S. cerevisiae* and *K. marxianus* in the fermentation of various lignocellulosic biomasses (Goshima et al. 2013, Yan et al. 2015). Nevertheless, the thermotolerance of *K. marxianus* and its ability to grow in high concentrations of a wide spectrum of sugars makes it of great interest for bioethanol production, and further studies should be carried out to take advantage of this yeast (Kim et al. 2019) (Table 2).

On the other hand, *O. polymorpha* is another yeast species with promising attributes for 2 G ethanol production. *Ogataea polymorpha* exhibits robust growth and fermentation capabilities under a wide range of environmental conditions, including temperatures as high as 45°C. This thermotolerance is advantageous for SSF processes, where lignocellulosic biomass is enzymatically hydrolyzed at elevated temperatures. However, ethanol production from xylose and other pentose sugars in *O. polymorpha* remains a challenge. Efforts are focused on optimizing metabolic pathways and genetic engineering to develop tailored strains that maximize ethanol production from lignocellulosic biomass while overcoming inherent metabolic limitations (Vasylyshyn et al. 2025) (Table 2).

It is important to note that genomic resources from diverse microorganisms, along with biological systems and mutagenesis approaches, have been utilized to engineer yeasts capable of fermenting lignocellulosic hydrolysates. As a result, several industrial and laboratory strains of *S. cerevisiae* capable of assimilating sugars derived from lignocellulosic hydrolysates through the heterologous expression of specific metabolic pathways have been developed. However, their efficiency still requires significant improvement to be suitable for cellulosic ethanol production at a large-scale industrial level. On the other hand, non-*Saccharomyces* strains represent a promising alternative for bioethanol production processes, as they naturally possess the ability to consume a broad spectrum of sugars, including the predominant pentoses and hexoses present in lignocellulosic hydrolysates. Nevertheless, these strains must exhibit several essential traits, such as genetic diversity, high ethanol productivity, thermotolerance, high ethanol concentration tolerance, and resistance to inhibitory compounds, among others. Therefore, selecting either *S. cerevisiae* or non-*Saccharomyces* strains for lignocellulosic bioethanol production ultimately depends on the targeted application, as well as the available technological and economic resources.

## Conclusions

This review compares *S. cerevisiae* and nonconventional yeasts for lignocellulosic ethanol production, highlighting their respective strengths and limitations. While *S. cerevisiae* is the industrial standard due to its robustness and ethanol tolerance, it requires substantial engineering for efficient pentose fermentation. In contrast, native pentose-fermenting yeasts such as *S. stipitis, K. marxianus*, and *O. polymorpha* offer broader substrate utilization and thermotolerance but face challenges with ethanol yield, inhibitor resistance, and oxygen dependence. By outlining these characteristics, the review provides guidance for strain selection and genetic improvement strategies, supporting the development of efficient and cost-effective microbial platforms for 2 G bioethanol production.
