# Correction to: Recent progress in engineering yeast producers of cellulosic ethanol

**DOI:** 10.1093/femsyr/foaf061

**Published:** 2025-10-10

This is a **correction** to:

Roksolana Vasylyshyn, Justyna Ruchala, Kostyantyn Dmytruk, Andriy Sibirny, Recent progress in engineering yeast producers of cellulosic ethanol, *FEMS Yeast Research*, Volume 25, 2025, foaf035, https://doi.org/10.1093/femsyr/foaf035.

In the originally published version of this manuscript, the funding acknowledgement is incomplete. According to the funder’s requirements, it should state:

“This work has received funding through the MSCA4Ukraine project (grant number 1232295), which is funded by the European Union. Views and opinions expressed are however those of the author(s) only and do not necessarily reflect those of the European Union. Neither the European Union nor the MSCA4Ukraine Consortium as a whole nor any individual member institutions of the MSCA4Ukraine Consortium can be held responsible for them. This work was partially supported by the Simons Foundation through the Presidential Discretionary—Ukraine Support Grants programme (grant number SFI-PD-Ukraine-00014576).”

This error has been corrected.

